# Prediction of the SF-6D utility score from Lung cancer FACT-L: a mapping study in China

**DOI:** 10.1186/s12955-023-02209-8

**Published:** 2023-11-14

**Authors:** Qing Yang, Long Lin Jiang, Yin Feng Li, Deyu Huang

**Affiliations:** 1 Nursing Department, Sichuan Clinical Research Center for Cancer, Sichuan Cancer Hospital & Institute, Sichuan Cancer Center, Affiliated Cancer Hospital of University of Electronic Science and Technology of China, 610041 Chengdu, China; 2 School of Nursing, Chengdu Medical College, 610500 Chengdu, China

**Keywords:** SF-6D · FACT-L, Mapping, Beta-mixture, Linear regression, Response mapping, Health-related quality of life (HRQoL)

## Abstract

**Objective:**

To develop a mapping algorithm for generating the Short Form Six-Dimension (SF-6D) utility score based on the Functional Assessment of Cancer Therapy-Lung (FACT-L) of lung cancer patients.

**Methods:**

Data were collected from 625 lung cancer patients in mainland China. The Spearman rank correlation coefficient and principal component analysis were used to evaluate the conceptual overlap between the FACT-L and SF-6D. Five model specifications and four statistical techniques were used to derive mapping algorithms, including ordinary least squares (OLS), Tobit and beta-mixture regression models, which were used to directly estimate health utility, and ordered probit regression was used to predict the response level. The prediction performance was evaluated using the correlations between the root mean square error (RMSE), mean absolute error (MAE), concordance correlation coefficient (CCC), Akaike information criterion (AIC) and Bayesian information criterion (BIC) and the observed and predicted SF-6D scores. A five-fold cross-validation method was used to test the universality of each model and select the best model.

**Results:**

The average FACT-L score was 103.024. The average SF-6D score was 0.774. A strong correlation was found between FACT-L and SF-6D scores (ρ = 0.797). The ordered probit regression model with the total score of each dimension and its square term, as well as age and sex as covariates, was most suitable for mapping FACT-L to SF-6D scores (5-fold cross-validation: RMSE = 0.0854; MAE = 0.0655; CCC = 0.8197; AEs > 0.1 (%) = 53.44; AEs > 0.05 (%) = 21.76), followed by beta-mixture regression for direct mapping. The Bland‒Altman plots showed that the ordered probit regression M5 had the lowest proportion of prediction scores outside the 95% agreement limit (-0.166, 0.163) at 4.96%.

**Conclusions:**

The algorithm reported in this paper enables lung cancer data from the FACT-L to be mapped to the utility of the SF-6D. The algorithm allows the calculation of quality-adjusted life years for cost-utility analyses of lung cancer.

**Supplementary Information:**

The online version contains supplementary material available at 10.1186/s12955-023-02209-8.

## Background

Lung cancer is the second most common and deadly cancer in the world, with GLOBOCAN estimating 2.2 million new cases and 1.8 million deaths worldwide in 2020 [1]. According to statistics from the National Cancer Center of China, the incidence and mortality of lung cancer rank first among malignant tumors in China [2]. The treatment of lung cancer patients imposes a huge economic burden on the Chinese health care system [3]. It is expected that the total economic burden of lung cancer will increase to 40.4 billion USD and 53.4 billion USD in 2025 and 2030, accounting for 0.131% and 0.146% of China’s GDP, respectively [3].

To rationally allocate limited health care resources, health economic evaluations are very important. Currently, the most commonly used cost-utility analysis (CUA) in health economic evaluations entails the use of quality-adjusted life-years (QALYs), which combine health-related quality of life (HRQoL) and survival into a single metric [4]. The QALY parameter, which can assess the health benefits of interventions not only across treatment strategies but also across patient groups, is recommended by health technology assessment (HTA) agencies such as the UK’s National Institute for Health and Clinical Excellence (NICE) [5]. Preference-based HRQoL tools are commonly used for QALY calculations, such as the EuroQol Five Dimensions (EQ-5D) and Short Form Six-Dimension (SF-6D). Although preference-based health status values would ideally be determined prospectively in clinical studies, this is not always the case [6, 7]. In clinical research, non-preference-based disease-specific questionnaires are often used because they capture more disease-specific or disease-related aspects from clinical and patient perspectives [8]. At this point, “mapping” (or “crosswalking”) [7, 9] can be used to address such problems; that is, in the case of trials that include disease-specific nonpreference measures of HRQoL but not preference-based measures, algorithms that allow the “transfer” of scores from disease-specific measures to utility measures can be generated.

Mapping includes direct mapping and indirect mapping. A previous systematic review found that the most commonly used method for mapping was ordinary least squares (OLS) for direct mapping, followed by Tobit regression [10]. Meanwhile, OLS has achieved the best predictive performance in multiple previous studies [11, 12]. Recent studies have demonstrated the potential value of mixed models to better capture the multimodal distribution of HRQoL data and manage the complexity of the data [13, 14]. In addition, mixed models can also capture changes in the impact of covariates on HRQoL in the distribution of HRQoL, such as the adjusted limited dependent variable mixed model (ALDVMM) [15] and beta-mixture regression model (BETAMIX) [16]. The method of indirect mapping has become increasingly popular in applied work [13], and related alternatives have been proposed, such as ordered logit or probit and generalized ordered probit models.

Currently, only two mapping studies have used non-preference-based quality of life scales (QLQ-C30, FACT-G) for the SF-6D and selected multiple cancer types, including lung cancer [17, 18]. In addition, some mapping studies on SF-6D have been carried out in other cancers, such as breast cancer [11] and thyroid cancer [19]. On the basis of Functional Assessment of Cancer Therapy-General (FACT-G), the Functional Assessment of Cancer Therapy-Lung (FACT-L) has added a dedicated module for lung cancer to measure HRQoL [11]. Because FACT-L is specifically designed for lung cancer patients and uses aspects that are significant for this specific patient population, the FACT-L is often the first choice in clinical studies. To date, however, no suitable method to convert the FACT-L score into a utility score is available to calculate the health utility value of lung cancer, which would facilitate economic evaluations of current or future lung cancer interventions. To address this gap, this study aimed to develop an algorithm to map the lung cancer-specific FACT-L score to the SF-6D utility score in lung cancer patients to promote CUA in a Chinese lung cancer patient population.

## Method

### Study design and patients

This study followed the Mapping to Preference-Based Measures Reporting Standards (MAPS) checklist proposed for instrumental mapping [20, 21] and the International Society for Pharmacoeconomics and Outcomes Research (ISPOR) reporting standards on mapping [9], as well as the systematic review of mapping research in the annual report of the National Institute for Health and Care Excellence (NICE) [22].

This cross-sectional study was designed to develop a mapping model from the FACT-L to the SF-6D. The study was conducted from October 2020 to November 2021 at the Sichuan Cancer Hospital, the largest tertiary first-class tumour hospital in Southwest China. The annual volume of thoracic surgery exceeds 4,000, and lung cancer patients from all over the country are admitted. The inclusion criteria for research subjects were as follows: (1) lung cancer diagnosed by clinical examination or pathological diagnosis; (2) age ≥ 18 years; (3) clear cognition, a normal demeanour, and a certain ability to understand and communicate; and (4) willingness to participate in this study with signing of the “Informed Consent”. Exclusion criteria: other serious chronic diseases, such as cardiovascular and cerebrovascular diseases and mental illness. Recruitment and informed consent procedures were performed by the investigators, and patients received questionnaires during their hospital outpatient visits or inpatient care. The ethics committee of Sichuan Cancer Hospital approved the conduct of this study (reference number: SCCHEC-02-2020-042). Written informed consent was obtained from all study participants.

### Research tool

#### FACT-L

The FACT-L (v.4.0) contains 36 questions in five dimensions, namely, 7 items for physical well-being (PWB), 7 items for social/family well-being (SWB), 6 items for emotional well-being (EWB), 7 items for functional well-being (FWB), and 9 additional items concerning lung cancer (LCS) (two of the nine were not included in the score) [23]. This system consists of a general scale (common module) FACT-G for measuring the common parts of the quality of life of cancer patients and an additional module for lung cancer. Among them, 27 items from the first 4 dimensions constitute FACT-G. Each item is scored between 0 and 4, and the total score of the scale is between 0 and 144, with higher scores indicating better quality of life. The Chinese version of the FACT-L (v4.0) has good reliability and validity [24].

### SF-6D

The SF-6D is a preference-based general health tool derived from the SF-36, including six dimensions of physical functioning, role limitations, social functioning, pain, mental health and vitality [25]. All dimensions consist of 4∽6 levels, creating a total of 18,000 possible health states [26]. In the absence of a utility algorithm for mainland China, we use the Chinese version (Hong Kong) of the SF-6D utility scoring system [27]. The scoring system has been demonstrated to be effective, with utility scores ranging between 0.315 and 1.

### Data collection

We asked participants to complete a combined questionnaire for both instruments. In addition to these two tools, we collected data on patient demographic and clinical characteristics. Data were collected by trained research team members. Before data collection, to ensure the quality of data collection, we also prepared a data collection manual.

### Exploratory data analysis—concept overlap

In this study, medians were used to fill individual missing values. SF-6D and FACT-L scores were assessed to test normality, skewness, and kurtosis using the skewness/kurtosis test before investigating concept overlap. Since the FACT-L and SF-6D scores were not normally distributed, this study used the Spearman correlation coefficient to calculate the overlap between the source and target instruments. Spearman’s rank correlation coefficient, which was defined before the analysis and used to interpret the results, ranks the strength of correlation into five levels—very weak (0–0.19); weak (0.20–0.39); moderate (0.40–0.59); strong (0.60–0. 0.79); and very strong (0.80–1.00) [28].

This study also employed principal component analysis (PCA) to explore and compare the underlying dimensional structure of the FACT-L data and the SF-6D information evident in these datasets [29]. The eigenvalue in PCA represents the amount of variance of the original variable explained by each principal component, which determines the number of principal components [30]. In this study, only principal components with eigenvalues greater than 1 were considered for the exploratory PCA, and rotation was performed using the tilted Promax method [29].

### Modelling methods and performance

We selected four models to map nonpreference-based health instruments onto general preference-based instruments: OLS, Tobit, ordinal probit regression (OPROBIT), and beta mixture regression (BETAMIX). Among them, OLS and Tobit regression are the two most widely used mapping methods [10, 20].

The utility score distribution obtained from general preference-based measures, such as the SF-6D, is usually not normally distributed and has a higher ceiling effect when the value is 1 and multiple peaks are present. Given that beta-mixture regression models have the advantage of addressing these issues [31], this study also used beta-mixture regression models to map the utility scores of the SF-6D. The beta-mixture regression model is a generalization of the truncated inflated beta regression model proposed by Botter Pereira [32]. Heterogeneity in the relationship between predictors and outcomes can be modelled by identifying clusters within the distribution, and mixture models provide a semiparametric and flexible approach to identifying these multimodalities [31, 33]. The beta-mixture regression model employed in this study specifies the gap between full health and the next feasible health state with a large number of observations at this cut-off point [34]. While the method could include probability mass at the lower bound of utility, we did not include this here because our sample did not contain any observations of the lower bound of utility for the SF-6D. We tried to use 1∽3 components to estimate the model, but some models may have convergence problems.

This study also used ordinal probit regression, which is an indirect method known as “response mapping”. Four to six separate equations were used to estimate the probability for each level of the different health domains covered by the SF-6D. From these regressions, expected health utility scores were then derived based on the set of health utility values. This approach has an intuitive appeal compared to linear regression because it is more closely related to the actual data generation process for health utility [13].

The model included the five domains of the FACT-L scale as well as age and sex as potential variables to predict the utility of the EQ-5D-5 L while also considering the squared terms of the total score and domain scores [28, 29]. Five modelling methods were used in each of the four models, and a two-tailed *p* value less than 0.05 was considered statistically significant.

Model 1: FACT-H&N total score.

Model 2: FACT-H&N total score + the square term of the FACT-H&N total score.

Model 3: Scores for each domain of FACT-H&N.

Model 4: Scores for each domain of FACT-H&N + the square term of the scores for each domain of FACT-H&N.

Model 5: Model 4 + age + sex.

Four common prediction performance indicators were used to evaluate the ability of candidate models to accurately predict SF-6D values: the root mean square error (RMSE), mean absolute error (MAE), and mean error (ME). These indicators are used as simple fitting summary measures and can be used for cross-model comparisons. In addition, this study also calculated Lin’s concordance correlation coefficient (CCC) to evaluate the relationship between the predicted and observed values of the FACT-L and SF-6D [35]. A CCC value close to 1 implies good agreement between the predicted and observed measurements.

Since the penalized likelihood measures (Akaike information criterion and Bayesian information criterion) cannot be used for direct comparisons of direct and indirect mapping models, they were used to select the best model in each class [36]. A Bland‒Altman analysis was performed by plotting the distribution of the difference between the observed and predicted utility (y-axis) and the mean of the observed and predicted utility (x-axis) [37]. In the process of preliminary selection and final screening of the best model, this study carried out a comprehensive ranking according to the average rank of each index [38]. This study examined the predictive performance of the SF-6D continuum model, the best of the various models, using cumulative distribution values. This study also used a Bland–Altman plot. The width between the 95% empirical limits of agreement was determined, which was compared with the width of the 95% theoretical limits of agreement.

In each model, we preliminarily selected the 2 best models and then used 5-fold cross-validation to assess the overfitting of the models and finally select the best model. This in-sample cross-validation technique randomly divides the initial dataset into 5 subsamples of uniform size. Four of the subsamples are used for parameter estimation, and one subsample is used for validation. The process was repeated five times, each subsample was used for only one validation, and the results were averaged to assess the overall performance of the model.

All statistical analyses were performed using Stata® version 15.0 (StataCorp LP, College Station, Texas, USA), except that R4.1.1 was used for CCC determination and PCA was performed in SPSS 23.0 (IBM Corp., Armonk, NY, USA). The Stata command “betamix” was used to fit the mixed regression model for the dependent variable in the interval [16], and the Stata command “oprobit” was used to fit the ordered probit regression model.

## Results

### Descriptive statistics

**Fig. 1:**
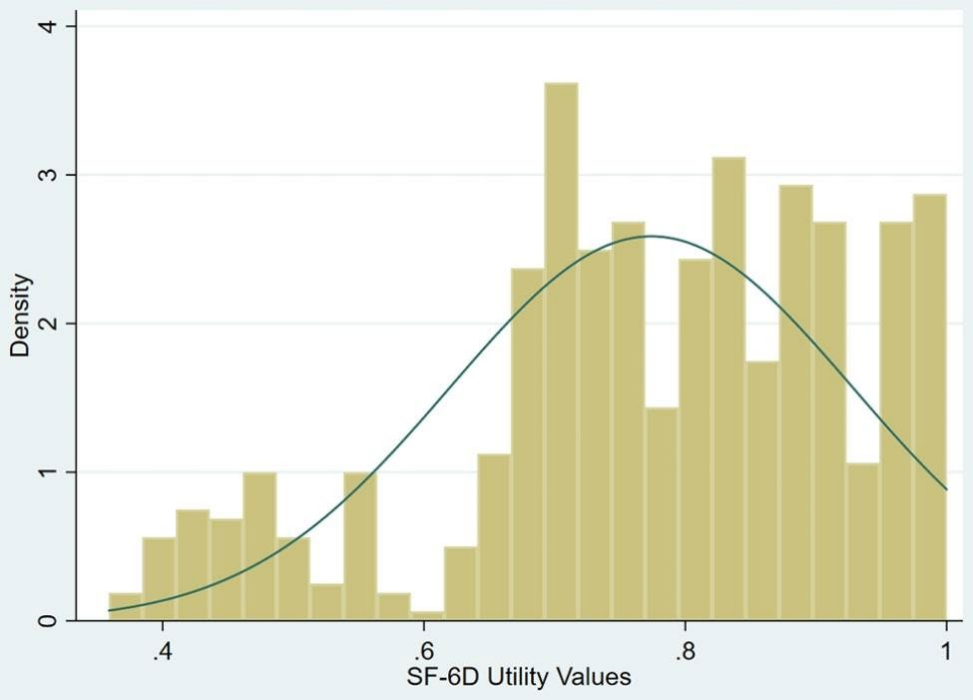
The distribution of SF-6D utility scores in the sample

Data from 625 lung cancer patients were collected for analysis and modelling in this study. In the demographic data, FACT-L, and SF-6D scales of 625 lung cancer patients, missing data accounted for 0.062%. Data analysis was conducted after imputing the missing values. Table 1 shows the characteristics and utilities of the study sample. The mean (standard deviation, SD) age of the patients, 45.8% of whom were women, was 58.290 (9.872) years old. A good representation of disease severity, in terms of cancer TNM stage, was observed in the study sample. FACT-L scores ranged from 54 to 133, with a mean of 103.024 (SD = 15.554), and were nonnormally distributed (Pr(Skewness) = 0.0006, Pr(Kurtosis) = 0.7092, *p* = 0.000). Figure 1 shows the distribution of the SF-6D scores, which was skewed to the right. The utility values of the SF-6D ranged from 0.359 to 1, with a mean of 0.774 (SD = 0.359), and showed a significantly right-skewed distribution (Pr(Skewness) = 0.0000, Pr(Kurtosis) = 0.7092, *p* = 0.000).

**Table 1:** Characteristics of the study sample

Variables	Mean (SD)	Min	Max
Utility measures			
SF-6D	0.774(0.154)	0.359	1
FACT-L			
Total scores	103.024(15.554)	54	133
PWB	22.464(5.028)	7	28
SWB	21.461(3.531)	0	28
EWB	17.483(3.281)	5	24
FWB	18.765(6.064)	2	28
LCS	22.851(3.109)	12	28
Socio-demographics			
Age	58.290(9.872)	24	82
Female, *n* (%)	286(45.8)		
TNM stage, *n* (%)			
I	284(45.44)		
II	73(16.68)		
III	130(20.8)		
IV	134(21.44)		
Unknown	4(0.64)		

### Overlap of concepts

Table 2 presents the Spearman rank correlation coefficients between the FACT-L and the total scores and domains of the SF-6D utility scores. Conceptually, the FACT-L score was positively correlated with the total SF-6D score (a higher FACT-L score is associated with a higher SF-6D utility score, indicating better health), the utility value of the SF-6D was positively correlated with the FACT-L total score and the scores for each dimension (higher FACT-L scores and higher SF-6D utility scores indicate better health), and SF-6D domain scores were negatively correlated with FACT-L total and domain scores (lower SF-6D domain scores correspond to higher FACT-L scores, indicating better health). Table 2 shows a strong correlation between the total scores of the SF-6D and FACT-L scales (ρ = 0.797); a negative correlation was found between each dimension, the correlation coefficient was − 0.217∽-0.758, and all correlation coefficients were statistically significant (*p* < 0.001). The correlation coefficient between the scores for each dimension of the FACT-L scale and the score for the SF-6D scale was highest in the PWB dimension and lowest in the SWB dimension.

Table 3 shows the PCA results, and the eight principal components explained 61.228% of the total variance. The first five principal components were the same as the SF-6D domains (“physical well-being”, “social/family well-being”, “emotional well-being”, “functional well-being”, and “LCS”), the sixth and seventh components can also be summarized into “physical well-being” and “LCS”, and the eighth component corresponds to “Sex/Weight”. Four SF-6D dimensions (“PF”, “RL”, “SF” and “PAIN”) were loaded to describe components related to the “physical well-being” dimension of the FACT-L. The other two SF-6D dimensions (“MH” and “VIT”) were loaded into “EWB”, “FWB” and “LCS”. None of the SF-6D dimensions were primarily loaded on the “SWB” component, which supports the poor correlation described above.

**Table 2:** Correlation between SF-6D scale and FACT-L scale scores

Dimension	PWB	SWB	EWB	FWB	HNCS	FACT-L total score
Physical functioning	-0.725^***^	-0.266^***^	-0.235^***^	-0.684^***^	-0.434^***^	-0.696^***^
Role limitations	-0.706^***^	-0.234^***^	-0.217^***^	-0.625^***^	-0.415^***^	-0.657^***^
Social functioning	-0.758^***^	-0.308^***^	-0.305^***^	-0.693^***^	-0.448^***^	-0.740^***^
Pain	-0.669^***^	-0.240^***^	-0.215^***^	-0.577^***^	-0.422^***^	-0.614^***^
Mental health	-0.423^***^	-0.337^***^	-0.507^***^	-0.404^***^	-0.347^***^	-0.539^***^
Vitality	-0.699^***^	-0.287^***^	-0.407^***^	-0.647^***^	-0.513^***^	-0.728^***^
SF-6D	0.810^***^	0.327^***^	0.354^***^	0.738^***^	0.515^***^	0.797^***^

^***^p<0.001

**Table 3:** Principal component analysis—rotated factor matrix

Instruments and items	Component							
	PWB	SWB	EWB	FWB	LCS	PWB2	LCS2	Sex/ Weight
PHYSICAL WELL-BEING(PWB)								
I have a lack of energy	0.786							
I have nausea						0.646		
I have trouble meeting the needs of my family	0.812							
I have pain	0.577						0.476	
I am bothered by side effects of treatment						0.607	0.443	0.430
I feel ill	0.761							
I am forced to spend time in bed	0.873							
SOCIAL/FAMILY WELL-BEING(SWB)								
I feel close to my friends		0.768						
I get emotional support from my family		0.830						
I get support from my friends		0.859						
My family has accepted my illness		0.869						
I am satisfied with family communication about my illness		0.776						
I feel close to my partner		0.609						
I am satisfied with my sex life								0.751
EMOTIONAL WELL-BEING(EWB)								
I feel sad			0.714					
I am satisfied with how I am coping with my illness				0.649				
I am losing hope in the fight against my illness			0.501					
I feel nervous			0.816					
I worry about dying			0.861					
I worry that my condition will get worse			0.830					
FUNCTIONAL WELL-BEING(FWB)								
I am able to work	0.831			0.584				
My work is fulfilling	0.844			0.597				
I am able to enjoy life	0.787			0.658				
I have accepted my illness	0.500			0.571				
I am sleeping well				0.550				
I am enjoying the things I usually do for fun	0.756			0.605				
I am content with the quality of my life	0.494			0.696				
LCS								
I have been short of breath					0.725			
I am losing weight								0.501
My thinking is clear							0.738	
I have been coughing					0.687			
I have a good appetite	0.544			0.439			0.408	
I feel tightness in my chest					0.675			
Breathing is easy for me	0.455							
SF-6D								
PF	0.853							
RL	0.826							
SF	0.869							
PAIN	0.733							
MH			0.661	0.414				
VIT	0.751		0.418	0.482			0.445	

### Model performance

Table 4 summarizes the performance of the models in the full sample (evaluated by seven goodness-of-fit indicators: the RMSE, MAE, CCC, AEs > 0.1 (%), AEs > 0.05 (%), AIC, and BIC) and sorts the best models according to the average rank of each indicator. Comparing the average ranks of different models in different mapping methods, Model 5 of OLS, Tobit, and ordered probit regression performed the best, followed by Model 4. Beta-mixture regression models without truncation performed better than those with truncation, and the average ranks of BETAMIX M4a and BETAMIX M4a were the lowest. Among all models, the RMSE of OLS M5 was the lowest (0.0838), the MAE of OLS M5 and BETAMIX M5a was the lowest (0.0653), the CCC of TOBIT M5 was the highest (0.8300), AEs > 0.1 (%) was lowest in TOBIT M5 and OPROBIT M5 (52.96%), and AEs > 0.05 (%) was lowest in TOBIT M3 (52.96%).

Table 5 shows the predicted values of the eight best candidate models. The OLS model best predicted the mean. The median predicted by the BETAMIX M4a model was closer to the observed value. Our best-fitting model overpredicted severe health states and underpredicted better health states.

**Fig. 2:**
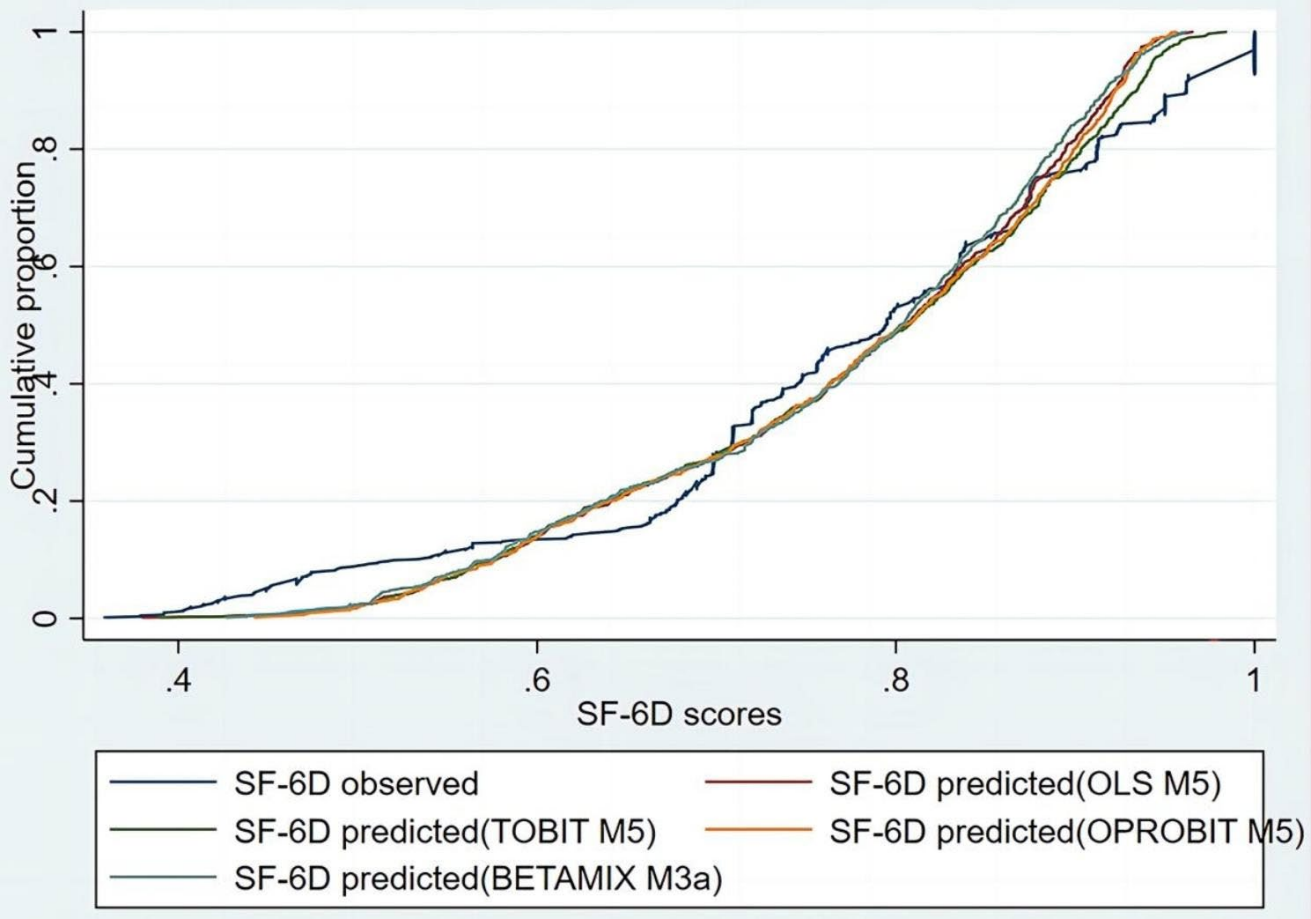
Cumulative distribution functions of observed and predicted SF-6D scores

**Fig. 3:**
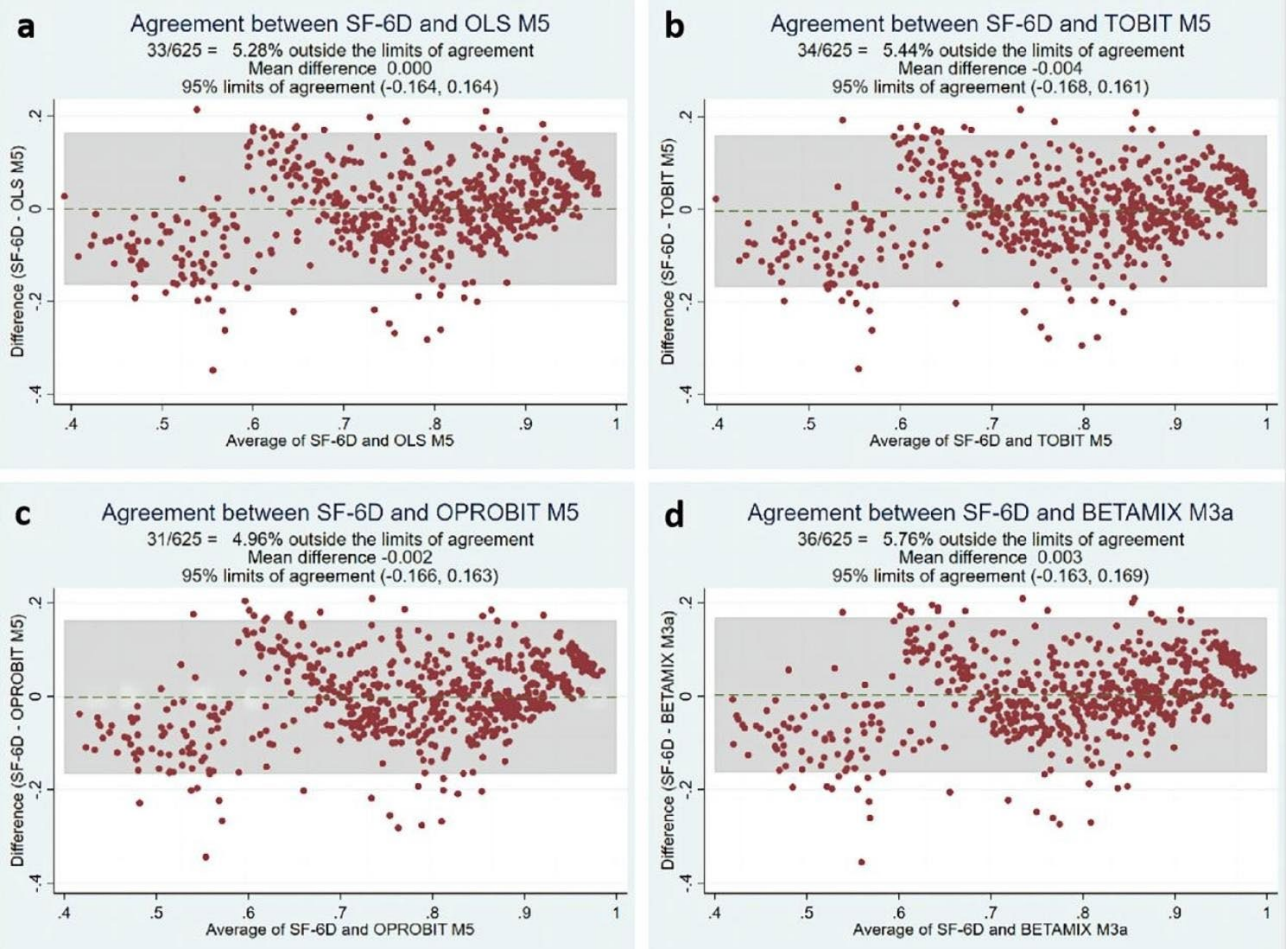
Bland–Altman plots of the observed and predicted SF-6D scores for **a** OLS M5, **b** TOBIT M5, **c** OPROBIT M5, **d** BETAMIX M3a

One of the best models was selected from the four modelling techniques to draw a conditional distribution function graph (Fig. 2), which showed that the predicted data of the SF-6D were in good agreement with the observed data. A Bland‒Altman analysis was performed to understand the predictive performance at the individual level. Figure 3 shows that the average residuals of the 4 best models were − 0.004∽0.003, with the average residual of OLS Model 5 being the smallest, followed by ordered probit regression. Ordered probit regression M5 had the lowest proportion of prediction scores outside the 95% agreement limit (-0.166, 0.163) at 4.96%.

Through 5-fold cross-validation to test the predictive ability of the eight best candidate models, the indicators of various predictive performances obtained from the verification samples were identified and are shown in Table 6. The average rank demonstrated that ordered probit Model 5 had the best comprehensive ranking (RMSE = 0.0854, MAE = 0.0655, CCC = 0.8197, AEs > 0.05 = 53.44%, AEs > 0.05 = 21.76%), followed by beta-mixture regression Model 3a (RMSE = 0.0853, MAE = 0.0661, CCC = 0.8194, AEs > 0.05 = 54.24%, AEs > 0.05 = 20.8%). The CCC between the observed utility and the predicted utility of the SF-6D obtained in the validation sample was 0.8185∽0.8230, indicating good agreement.

**Table 4:** Model performance of four regression methods that map FACT- L to SF- 6D utility scores

No	Mapping	RMSE	MAE	CCC	AE > 0.1	AE > 0.05	AIC	BIC	ARV
	method				(%)	(%)			
1	OLS M1	0.0977	0.0764	0.7482	58.24	29.92	-1129.08	-1120.20	4.64
2	OLS M2	0.0977	0.0763	0.7485	57.92	29.44	-1127.63	-1114.31	4.36
3	OLS M3	0.0849	0.0658	0.8211	55.20	21.28	-1297.23	-1270.60	2.50
4	OLS M4	0.0841	0.0654	0.8251	53.12	21.60	-1299.07	-1250.25	2.00
5	OLS M5	0.0838	0.0653	0.8265	53.60	21.28	-1299.42	-1241.73	1.50
6	TOBIT M1	0.0980	0.0763	0.7558	57.76	30.08	-888.24	-874.92	4.14
7	TOBIT M2	0.0981	0.0765	0.7555	57.92	29.92	-886.38	-868.63	4.86
8	TOBIT M3	0.0851	0.0658	0.8250	54.08	19.84	-1035.70	-1004.64	2.43
9	TOBIT M4	0.0844	0.0655	0.8286	53.60	21.44	-1037.33	-984.08	1.93
10	TOBIT M5	0.0841	0.0655	0.8300	52.96	21.60	-1038.11	-975.99	1.64
11	OPROBIT M1	0.0975	0.0761	0.7519	59.20	28.96	8089.15	14211.95	4.86
12	OPROBIT M2	0.0973	0.0760	0.7529	57.60	29.44	8088.51	14151.67	4.14
13	OPROBIT M3	0.0852	0.0662	0.8204	53.92	20.32	7570.70	13350.07	2.57
14	OPROBIT M4	0.0843	0.0657	0.8245	54.40	21.92	7567.43	13290.18	2.00
15	OPROBIT M5	0.0839	0.0655	0.8262	52.96	21.76	7568.51	13309.17	1.43
	Beta-mixture regression models without truncation								
16	BETAMIX M1a	0.0973	0.0768	0.7500	58.24	30.24	-476.33	-454.14	9.93
17	BETAMIX M1b	0.0980	0.0724	0.7312	59.36	29.76	-500.06	-460.12	10.00
18	BETAMIX M2a	0.0973	0.0721	0.7506	58.56	29.60	-474.50	-443.44	9.50
19	BETAMIX M3a	0.0847	0.0657	0.8225	54.40	21.12	-628.08	-570.39	3.64
20	BETAMIX M3b	0.0848	0.0661	0.8217	54.24	21.28	-665.21	-572.02	4.07
21	BETAMIX M3c	0.0850	0.0663	0.8219	54.88	21.92	-672.91	-544.21	5.00
22	BETAMIX M4a	0.0842	0.0654	0.8250	53.92	21.12	-620.49	-518.42	2.86
23	BETAMIX M5a	0.0948	0.0653	0.8265	54.08	21.76	-624.50	-504.69	3.86
	Beta-mixture regression models with truncation								
24	BETAMIX M1a^#^	0.1585	0.1213	0.3480	72.16	47.84	155.21	168.52	13.43
25	BETAMIX M2a^#^	0.1585	0.1211	0.3480	72.00	47.68	154.95	172.70	13.00
26	BETAMIX M2b^#^	0.0968	0.0763	0.7549	58.72	29.44	-542.86	-489.61	8.43
27	BETAMIX M3a^#^	0.0851	0.0660	0.8226	54.24	20.96	-589.12	-531.43	4.50
28	BETAMIX M4a^#^	0.1538	0.1206	0.3992	75.68	48.48	44.94	98.21	12.57
29	BETAMIX M5a^#^	0.0843	0.0656	0.8263	53.92	22.40	-589.44	-469.62	4.21

^#^Beta-mixture regression models with truncation

**Table 5:** Descriptive summary of SF-6D utility index derived from observed and predicted values of best fitting models

Model	Mean	SD	Minimum	P10	Median	P90	Maximum
Observed data	0.7740	0.1542	0.3590	0.5340	0.7950	0.9620	1.0000
OLS M4	**0.7740**	0.1292	0.3801	0.5794	0.8047	0.9206	0.9578
OLS M5	**0.7740**	0.1294	**0.3797**	0.5777	0.8052	0.9218	0.9653
TOBIT M4	0.7777	0.1334	0.3899	0.5764	0.8057	0.9323	0.9746
TOBIT M5	0.7777	**0.1337**	0.3894	0.5753	0.8077	**0.9344**	**0.9840**
OPROBIT M4	0.7757	0.1294	0.4439	0.5751	0.8048	0.9239	0.9526
OPROBIT M5	0.7757	0.1296	0.4429	0.5765	0.8033	0.9224	0.9561
BETAMIX M3a	0.7714	0.1292	0.4268	0.5756	0.8028	0.9173	0.9617
BETAMIX M4a	0.7714	0.1296	0.4089	**0.5713**	**0.8006**	0.9148	0.9647

The closest fit values to the observed data are highlighted in bold

**Table 6:** Goodness of Fit results of the best fitting model validation analysis: 5-fold cross-validation

	(1)RMSE	(2)MAE	(3)CCC	(4) AE > 0.1 (%)	(5)AE > 0.05 (%)	ARV
OLS M4	0.0856	0.0665	0.8186	53.60	22.08	3.9
OLS M5	0.0854	0.0665	0.8194	54.24	22.40	4.3
TOBIT M4	0.0859	0.0666	0.8222	54.56	22.24	5.2
TOBIT M5	0.0857	0.0667	**0.8230**	54.08	22.40	4.6
OPROBIT M4	0.0857	0.0667	0.8185	54.72	22.56	6.9
OPROBIT M5	0.0854	**0.0655**	0.8197	**53.44**	21.76	**2.1**
BETAMIX M3a	**0.0853**	0.0661	0.8194	54.24	20.8	2.8
BETAMIX M4a	0.0858	0.0667	0.8176	54.88	**18.88**	6.2

The best results among the mapping models are highlighted in bold

### Regression results

The regression coefficients of the OLS and Tobit models are shown in Additional files 1–2 (see Additional files 1–2), and the regression coefficients of ordered probit Model 5 and beta-mixture regression Model 3 are shown in Tables 7 and 8. In the OLS and Tobit models, the FACT-L total score and the coefficient of the square term of the total score were both positive and statistically significant (*p* < 0.05). FWB and PWB were significant positive predictors of SF-6D scores in the OLS and Tobit models. In addition, the squared term of LCS was an important predictor of SF-6D scores in the OLS and Tobit models after adding the squared term of each domain of the FACT-L. In addition to FWB and PWB, the EWB domain score was also an important predictor in some domains of ordered probit models. Considering two sociodemographic variables, namely, age and sex, only sex was statistically significant in the Tobit model and the ordered probit model (*p* < 0.05), and a significant positive correlation was identified between male sex and the utility score of the SF-6D. This study also provides Excel calculators for the best fit direct mapping model and indirect mapping model (see Additional files 3–4), which allows users to easily calculate SF-6D from FACT-L scores.

**Table 7:** Coefficient estimates of ordered probit regression: Model 5

Variable	SF-6D dimensions					
	Physical functioning	Role limitation	Social functioning	Pain	Mental health	Vitality
PWB	-0.10035	0.04258	0.02495	0.09956	0.14086	-0.11093
SWB	-0.08415	-0.11853	-0.06716	0.01935	-0.07695	-0.16562^*^
EWB	0.19117^*^	0.07062	0.04289	0.03117	-0.28455^**^	-0.05456
FWB	-0.10340^*^	-0.15476^**^	-0.09566^*^	0.00054	0.02716	-0.07315
LCS	0.39364^**^	0.07531	0.04314	0.06599	-0.01363	0.11623
Dimension squared						
PWB squared	-0.00187	-0.00533^*^	-0.00529^*^	-0.00611^***^	-0.00397^*^	-0.00112
SWB squared	0.00243	0.00319	0.00138	-0.00028	0.00037	0.00411^*^
EWB squared	-0.00433	-0.00082	-0.00072	0.00001	0.00308	-0.00015
FWB squared	0.00040	0.00259	0.00038	-0.00117	-0.00148	0.00010
LCS squared	-0.00909^**^	-0.00205	-0.00116	-0.00245	-0.00078	-0.00508
Age	0.00696	0.00513	0.00562	0.00101	-0.00865	0.00099
Gender	-0.33676^***^	-0.07934	-0.08691	-0.05482	0.01211	0.09509
/cut1	-1.03870	-3.31164	-4.07685	-1.03600	-6.57599	-7.42327
/cut2	0.15058	-2.27073	-2.83700	-0.39104	-4.49802	-4.80170
/cut3	0.84777	-1.16103	-2.02446	1.04159	-3.12214	-3.77475
/cut4	1.75682		-1.08890	1.85376	-1.84889	-2.11280
/cut5	3.01388			2.54350		

^*^p<0.05, ^**^p<0.01, ^***^p<0.001

**Table 8:** Coefficient estimates of Beta-mixture model: Model3a

sf6d	Coef.	Std. Err.	z	*p* > z	[95% Conf.	Interval]
C1_mu						
PWB	0.10784	0.00711	15.18000	0.00000	0.09391	0.12176
SWB	-0.00165	0.00750	-0.22000	0.82600	-0.01636	0.01306
EWB	0.00100	0.00825	0.12000	0.90300	-0.01518	0.01718
FWB	0.05047	0.00584	8.64000	0.00000	0.03902	0.06192
LCS	0.01068	0.00951	1.12000	0.26200	-0.00797	0.02932
_cons	-2.88634	0.23107	-12.49000	0.00000	-3.33922	-2.43346
C1_lnphi						
_cons	2.54900	0.05723	44.54000	0.00000	2.43682	2.66118
PM_ub						
PWB	0.61565	0.15424	3.99000	0.00000	0.31333	0.91796
SWB	0.09495	0.07471	1.27000	0.20400	-0.05148	0.24139
EWB	0.00483	0.05909	0.08000	0.93500	-0.11099	0.12065
FWB	0.14810	0.05836	2.54000	0.01100	0.03373	0.26248
LCS	0.19941	0.08247	2.42000	0.01600	0.03777	0.36104
_cons	-29.13472	4.67931	-6.23000	0.00000	-38.30600	-19.96343
C1_phi	12.79427	0.73228			11.43661	14.31311

## Discussion

This is the first study to produce a FACT-L to SF-6D mapping algorithm, and the availability of this algorithm means that researchers can obtain SF-6D health utility values for lung cancer patients simply by including the FACT-L even if the preference-based measurement tool itself is not included in the study. The final model included the total score for each dimension of the FACT-L, the squared term of the total score for the dimension, and sex. We found that models using subscales as independent variables outperformed models using total scores as independent variables [39]. All variables finally included were independent of the SWB dimension measured by the FACT-L, and similar results have been found in previous studies of other cancers [40]. On the one hand, this study found through correlation analysis that the correlation coefficient between the scores for each dimension of the FACT-L scale and the score for the SF-6D scale was the lowest in the SWB dimension. Conceptual differences between the source and target instruments may be another explanation, and exploratory factor analysis (EFA) is often used to examine the degree of conceptual overlap between the two, where PCA has been recommended as the preferred method for factor extraction [41]. From the PCA results of this study, none of the SF-6D dimensions were mainly loaded on the “SWB” component, which supports the result that the SWB dimension in the FACT-L scale cannot be entered into the mapping model.

Age and gender were considered important during the mapping process, and their inclusion was recommended where feasible [9]. Although previous mapping studies have added covariates such as the tumor stage [42] and Charlson comorbidity index [43], considering that other disease related variables may not be included in the study when using the algorithm of this study in the future, this study mainly considers age and gender in demographic variables. Among the four models in this study, age was not a statistically significant predictor of SF-6D scores, while sex was statistically significant in Tobit regression and ordered probit regression (*p* < 0.05). Male sex was significantly positively correlated with health utility scores [44]. The squared terms of LCS were significant predictors of SF-6D scores in both OLS and Tobit models after adding the squared terms in each domain of the FACT-L. The addition of the square term is beneficial to improve the performance of the model [44, 45].

This study adopted internal validation, passed five indicators [46], and found that ordered probit Model 5 was the best model for comprehensive ranking (RMSE = 0.0854, MAE = 0.0655, CCC = 0.8197, AEs > 0.1 = 53.44%, AEs > 0.05 = 21.76%), followed by beta-mixture regression Model 3a (RMSE = 0.0853, MAE = 0.0661, CCC = 0.8194, AEs > 0.1 = 54.24%, AEs > 0.05 = 20.8%), which is consistent with recent evidence indicating that indirect mapping and mixed models are better than linear regression. In this study, the difference between the SF-6D value obtained by all the best prediction models and the observed value was greater than 0.10 (absolute error percentage > 0.10) in less than 60% of the samples, which is lower than those in previous mapping studies [43].

In addition, good agreement was noted between the predicted value of the SF-6D and the measured value in this study because most of the observed values fell in the mean ± 1.96 SD difference area, and the Bland‒Altman plot showed that the predicted score of ordered probit M5 exceeds 95%. The consistency limit (-0.166, 0.163) had the lowest proportion (4.96%), and this result is similar to the results of previous mapping models [47]. The CCC (0.8185 ∽ 0.8230) between the observed and predicted utility of the SF-6D obtained in the validation sample was higher than 0.8, which is slightly higher than those in previous studies [48]; this is a good result and highlights the prediction. Good agreement was observed between the values and observations [49]. Past studies have shown that mapping is more likely to be successful if two tools overlap conceptually [50, 51]. Before mapping, this study explored the strong correlation between the total scores of the two scales through Spearman rank correlation analysis (ρ = 0.797), and all the correlation coefficients between the total score and the scores for each dimension were statistically significant (*p* < 0.001). In addition, the PCA results also suggest that the SF-6D dimension is loaded on the components of the FACT-L scale except for “SWB”.

Overall, the mapping function performed well in this study. While our best-fitting models overpredicted severe health states, they underpredicted better health states [8, 48]. For example, although the 10th percentile (0.5713) predicted by BETAMIX M4a for the SF-6D was closest to the observed value of 0.5340, it was above the observed value. Although the mapping model approaches used are not completely consistent, this finding is similar to previous research results [29]. Although the conditional distribution function plots show that the simulated SF-6D data from the best-fitting model are in good agreement with the observed data, these plots also confirm that these models generally underestimate the value of the SF-6D for mildly healthy states and overestimate the value of the SF-6D for healthier states [52]. At the same time, the Bland–Altman plot in this study also shows this difference between the observed and predicted values, which is similar to results in published literature related to mapping models [37]. This “mismatch” is a common problem in mapping studies and is mainly due to regression to the mean.

This study has some strengths. Given the lack of international studies and the absence of studies in China, this study developed a mapping algorithm to predict the SF-6D utility score in lung cancer patients. Second, this study utilized four regression models and five evaluation criteria to determine the best-performing algorithm while employing and comparing direct and indirect mapping methods.

### Limitations of this study

Although the lung cancer patients in this study had different stages and pathological types, our sample size was limited to a single sample, and the results may not be representative of the entire lung cancer population in China. Future work could include data on lung cancer patients from more regions in China. Second, no other independent datasets with SF-6D and FACT-L observations were available to assess the external validity of the mapping algorithm reported in this study. Therefore, we did not use other datasets to evaluate the external validity of the mapping algorithm in this study, which may limit the generalizability of its application to some extent.

## Conclusions

In conclusion, the FACT-L can be mapped to SF-6D utility with good prediction accuracy. Ordered probit regression models were best suited for mapping FACT-L scores to SF-6D scores, followed by beta-mixture regression for direct mapping. Our mapping algorithm can compute QALYs when no preference-based health utility measure is available for lung cancer patients. It can compare the cost-effectiveness of lung cancer-related interventions and help relevant decision-makers reach scientific decisions regarding the allocation of limited resources.

### Electronic supplementary material

Below is the link to the electronic supplementary material.

Supplementary Material 1

Supplementary Material 2

Supplementary Material 3

Supplementary Material 4

## Data Availability

The datasets are available from the corresponding author on reasonable request.

## Abbreviations

AE: Absolute error
AIC: Akaike Information Criterion
ALDVMM: Adjusted limited dependent variable mixed model
BETAMIX: Beta-mixture regression model
BIC: Bayesian information criterion
CCC: concordance correlation coefficient
CUA: Cost-utility analysis
EFA: Exploratory factor analysis
EQ-5D: EuroQol-5 dimension
EWB: Emotional well-being
FACT-L: Functional Assessment of Cancer Therapy- Lung
FWB: Functional well-being
HRQoL: Health-related quality of life
HTA: Health technology assessment
ISPOR: International Society for Pharmacoeconomics and Outcomes Research
LCS: Additional concerns about lung cancer
MAE: Mean absolute error
MAPS: Mapping onto Preference-Based Measures Reporting Standards
NICE: National Institute for Health and Clinical Excellence
OLS: Ordinary least-square
OPROBIT: Ordinal probit regression
PCA: Principal component analysis
PWB: Physical well-being
QALYs: Quality-adjusted life years
RMSE: Root mean squared error
SF-6D: Short Form Six-Dimension
SWB: social/family well-being
