# Correction: Prediction of the SF-6D utility score from lung cancer FACT-L: a mapping study in China

**DOI:** 10.1186/s12955-024-02237-y

**Published:** 2024-02-27

**Authors:** Qing Yang, Long Lin Jiang, Yin Feng Li, Deyu Huang

**Affiliations:** 1 Nursing Department, Sichuan Clinical Research Center for Cancer, Sichuan Cancer Hospital & Institute, Sichuan Cancer Center, Affiliated Cancer Hospital of University of Electronic Science and Technology of China, 610041 Chengdu, China; 2 School of Nursing, Chengdu Medical College, 610500 Chengdu, China

Health and Quality of Life Outcomes (2023) 21:122

10.1186/s12955-023-02209-8

The original article contains minor text errors in the Modelling methods and performance sub-section of the Methods sections.

The following sentences should each state ‘FACT-L’ in place of each instance of ‘FACT H&N’:

Model 1: FACT-H&N total score.

Model 2: FACT-H&N total score + the square term of the FACT-H&N total score.

Model 3: Scores for each domain of FACT-H&N.

Model 4: Scores for each domain of FACT-H&N + the square term of the scores for each domain of FACT-H&N.

