# Supplementary material for: Prediction of the SF-6D utility score from Lung cancer FACT-L: a mapping study in China
Source: Health Qual Life Outcomes. 2023 Nov 14;21:122. doi: 10.1186/s12955-023-02209-8 (PMC10648360; doi:10.1186/s12955-023-02209-8)
Supplement: Supplementary file 1 — Supplementary Material 1 [file 12955_2023_2209_MOESM1_ESM.docx]

Additional file 1

Coefficient estimates of ordinary least-square regression

| Variable | OLS M1 | OLS M2 | OLS M3 | OLS M4 | OLS M5 |
| --- | --- | --- | --- | --- | --- |
| Constant | -0.01578 | -0.12171 | 0.19682^***^ | 0.47883^***^ | 0.50053^***^ |
| FACT-L total score | 0.00767^***^ | 0.00983* |  |  |  |
| FACT-L squared |  | -0.00001 |  |  |  |
| PWB |  |  | 0.01754^***^ | 0.01231^*^ | 0.01205^*^ |
| SWB |  |  | 0.00024 | 0.00600 | 0.00668 |
| EWB |  |  | -0.00047 | -0.00881 | -0.00872 |
| FWB |  |  | 0.00767^***^ | 0.01401^***^ | 0.01361^***^ |
| LCS |  |  | 0.00185 | -0.02342^*^ | -0.02383^*^ |
| Dimension squared |  |  |  |  |  |
| PWB squared |  |  |  | 0.00013 | 0.00014 |
| SWB squared |  |  |  | -0.00015 | -0.00017 |
| EWB squared |  |  |  | 0.00026 | 0.00026 |
| FWB squared |  |  |  | -0.00019^*^ | -0.00018 |
| LCS squared |  |  |  | 0.00058^*^ | 0.00059^*^ |
| Age |  |  |  |  | -0.00039 |
| Gender |  |  |  |  | 0.01321 |

^*^ *P*＜0.05，^**^ *P*＜0.01，^***^ *P*＜0.001
