# Supplementary material for: Prediction of the SF-6D utility score from Lung cancer FACT-L: a mapping study in China
Source: Health Qual Life Outcomes. 2023 Nov 14;21:122. doi: 10.1186/s12955-023-02209-8 (PMC10648360; doi:10.1186/s12955-023-02209-8)
Supplement: Supplementary file 2 — Supplementary Material 2 [file 12955_2023_2209_MOESM2_ESM.docx]

Additional file 2

Coefficient estimates of Tobit model

| Variable | TOBIT M1 | TOBIT M2 | TOBIT M3 | TOBIT M4 | TOBIT M5 |
| --- | --- | --- | --- | --- | --- |
| Constant | -0.04841 | 0.00908 | 0.17096^***^ | 0.52630^***^ | 0.54924^***^ |
| FACT-L total score | 0.00803^***^ | 0.00315^*^ |  |  |  |
| FACT-L squared |  | 0.00001 |  |  |  |
| PWB |  |  | 0.01767^***^ | 0.00980 | 0.00947 |
| SWB |  |  | 0.00062 | 0.00611 | 0.00688 |
| EWB |  |  | -0.00047 | -0.00810 | -0.00800 |
| FWB |  |  | 0.00808^***^ | 0.01255^***^ | 0.01210^***^ |
| LCS |  |  | 0.00232 | -0.02620^*^ | -0.02662^*^ |
| Dimension squared |  |  |  |  |  |
| PWB squared |  |  |  | 0.00020 | 0.00021 |
| SWB squared |  |  |  | -0.00015 | -0.00018 |
| EWB squared |  |  |  | 0.00024 | 0.00024 |
| FWB squared |  |  |  | -0.00014 | -0.00013 |
| LCS squared |  |  |  | 0.00065^*^ | 0.00066^*^ |
| Age |  |  |  |  | -0.00042 |
| Gender |  |  |  |  | 0.01493^*^ |

^*^ *P*＜0.05，^**^ *P*＜0.01，^***^ *P*＜0.001
